# Brugada Syndrome: A Comprehensive Review of Fundamental and Electrophysiological New Findings

**DOI:** 10.3390/jcm12206590

**Published:** 2023-10-18

**Authors:** Naoya Kataoka, Teruhiko Imamura

**Affiliations:** Second Department of Internal Medicine, University of Toyama, 2630 Sugitani, Toyama 930-0194, Japan; nkataoka@med.u-toyama.ac.jp

**Keywords:** Brugada syndrome, conduction disorder, phase 2 reentry, ventricular fibrillation

## Abstract

Brugada syndrome is characterized by pronounced J-ST segment elevation in the right precordial leads on surface electrocardiograms. The etiological underpinnings of these distinctive features have been the subject of extensive debate, encompassing various theories related to repolarization anomalies and conduction irregularities. Genetic investigations have unveiled SCN5A, the gene encoding NaV1.5, a critical sodium channel, as the most frequently implicated causative gene, with mutations typically manifesting as loss of function. Nonetheless, the detection rate of SCN5A mutations remains below 20%, underscoring the intricate genetic landscape of the syndrome. Histological analyses have divulged localized structural irregularities, primarily marked by fibrotic alterations, within the right ventricular outflow tract. Electrophysiological inquiries employing direct epicardial mapping techniques have uncovered localized conduction impediments concomitant with modifications in unipolar morphologies within the J-ST segment. Thus, the theory positing conduction abnormalities emerges as a compelling mechanism accounting for J-ST segment elevation. However, the precise mechanisms governing the onset of life-threatening tachyarrhythmias remain shrouded in uncertainty. Recent clinical case reports have proffered evidence supporting the notion that phase 2 reentry, arising from the marked heterogeneity in action potentials within the epicardial domain, may serve as the instigator of premature ventricular contractions, ultimately culminating in ventricular fibrillation. In light of these developments, it becomes increasingly evident that comprehending the mechanisms underlying the electrocardiographic manifestations and lethal arrhythmias in Brugada syndrome necessitates the consideration of a multifaceted perspective, transcending the binary discourse of repolarization versus depolarization anomalies.

## 1. Introduction

Brugada syndrome (BrS), originally elucidated by the Brugada siblings in 1992, manifests as J-ST segment elevation discernible in the right precordial leads of the surface electrocardiogram (ECG). This electrocardiographic anomaly occurs in the absence of any underlying cardiac pathologies [1]. A minority of individuals afflicted by BrS experience malignant arrhythmias, yet their incidence is notably rare, estimated at 8–10% annually among patients with a history of ventricular fibrillation (VF), 0.5–2% annually in cases marked by syncope, and a mere 0–0.5% annually in asymptomatic cohorts [2,3]. Although the utilization of implantable cardioverter-defibrillators for patients who have experienced VF episodes enjoys widespread acceptance, a compelling exigency persists for an effective risk stratification approach for the vast majority who remain devoid of symptoms throughout their lifetime. Consequently, there exists an imperative to elucidate the intricacies of the Brugada-type ECG pattern and its association with the genesis of tachyarrhythmia.

Over the past three decades, the discourse surrounding J-ST elevation and the spontaneous occurrence of ventricular fibrillation (VF) has revolved around two prominent theories: the conduction disorder theory and the repolarization abnormality theory. In the realm of surface electrocardiography (ECG), specific markers of spontaneous VF, congruent with the conduction disorder theory, include QRS wave fragmentation during sinus rhythm, fragmentation within the terminal segment of the QRS during right ventricular apical pacing, a conspicuous prominence of the S-wave in lead I, and the extension of the PR interval. These markers are well-aligned with the conduction disorder theory [4,5,6].

Conversely, the repolarization abnormality theory is buttressed by risk factors such as the lengthening of the T peak-to-end interval, the presence of T-wave alternans, and concurrent early repolarization [7,8,9]. In experimental models, the current-to-load mismatch theory has proven to be a cogent framework elucidating the mechanisms underpinning ST-segment elevation in the context of conduction disorders [10]. In stark contrast, the concept of a heterogeneous loss of the action potential dome, propounded by Antzelevitch C., has gained widespread acceptance as the foundational mechanism underpinning ST-segment elevation [11].

These noninvasive ECG observations and experimental paradigms have proficiently elucidated the complex interplay between ECG characteristics and the genesis of spontaneous arrhythmias. Consequently, the ongoing discourse centers on the debate between these two theories pertaining to J-ST elevation, specifically the conduction disorder theory and the repolarization abnormality theory.

Recent studies have forged a novel avenue for comprehending genotypic and phenotypic anomalies via the employment of genetic testing and invasive electrophysiological investigations conducted on the epicardium. In this paper, we have undertaken a comprehensive review of contemporary discoveries emanating from the realms of genetics, histology, and epicardial electrophysiology. In this review, we aim to elucidate the underpinning mechanisms of BrS, thereby transcending the longstanding dichotomy between conduction disorder and repolarization abnormality theories.

## 2. Genetic Findings

**Figure 1:**
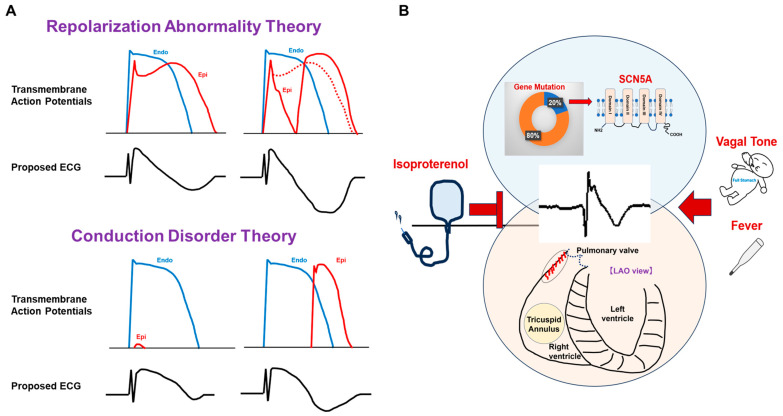
Theories of J-ST elevation and factors influencing Brugada syndrome. (**A**) The theory positing repolarization abnormality posits that the preservation of deep membrane potentials during phase 2, along with the extension of action potentials in the epicardium compared to those observed in the endocardium, precipitates J-ST elevation coupled with a negatively deflected T-wave. The absence of a prominent dome in epicardial action potentials instigates phase 2 reentry, driven by transmural dispersion, thereby exacerbating the manifestation of a negative T-wave. On the other hand, the theory emphasizing conduction disturbances posits that localized epicardial excitation failure underlies J-ST elevation, and delayed conduction culminates in the appearance of a negatively oriented T-wave, typically exhibiting the coved-type morphology. (**B**) Gene mutations, frequently affecting SCN5A, or structural cardiac pathologies, often characterized by localized fibrotic alterations in the right ventricular outflow tract and, at times, a combination thereof, serve as the genesis for the distinctive Brugada-type ECG patterns. Perturbations such as isoproterenol administration, vagal tone modulation, or elevated body temperature due to fever can exert influence over J-ST segment levels and the propensity for arrhythmic events.

BrS has been postulated as a primary ion channel disorder characterized by a relative attenuation of the inward sodium current compared to the transient outward potassium current, denoted as Ito, predominantly observed in the epicardium of the right ventricular outflow tract [12]. This derangement in ion currents culminates in transmural dispersion of the action potential duration, subsequently yielding J-ST elevation on the surface electrocardiogram (Figure 1A) [13]. It is worth noting that transmural dispersion of the action potential duration is recognized as a contributory factor to the genesis of ventricular tachyarrhythmias, not solely in the context of BrS but also in the setting of cardiac failure [14].

In the year 1992, coinciding with the inaugural report on BrS, SCN5A, the gene encoding sodium channel alpha subunits NaV1.5, was initially unveiled as the pathogenic mutation underlying BrS [15]. Within the repertoire of voltage-gated sodium channel family members, NaV1.1, NaV1.2, NaV1.3, NaV1.6, and NaV2.1 predominantly find expression in the central nervous system, while NaV1.7, NaV1.8, and NaV1.9 reside within the peripheral nervous system. NaV1.4 is situated within skeletal muscle, whereas NaV1.5 exclusively assumes the role of the principal sodium channel within the cardiac milieu [16]. Extensive genetic inquiries have revealed that mutations within SCN5A not only correlate with BrS but also extend their influence on Long QT syndrome, conduction disorders, and dilated cardiomyopathy. These variations emanate from the versatility of SCN5A mutations, which can precipitate both loss and gain of channel function. To date, it has been unequivocally established that loss-of-function mutations represent a pivotal factor in the clinical manifestation of the Brugada phenotype [17]. Furthermore, it is noteworthy that individuals harboring SCN5A mutations exhibit a heightened predisposition to spontaneous VF events when juxtaposed with their mutation-free counterparts [18].

While it is established that mutations within SCN5A represent the most frequently encountered genetic anomalies associated with BBrS, it is imperative to note that the detection rate for such mutations remains conspicuously below 20%, underscoring the intricate genetic heterogeneity intrinsic to the syndrome [12]. Notably, an intriguing case arose wherein a mutation within SCN5A, identified in a patient with BrS, failed to elicit the Brugada-type ECG pattern when assessed in a transgenic pig model [19]. Furthermore, administration of sodium channel blockers did not induce the Brugada-type ECG in any of the individuals harboring SCN5A mutations [20]. These revelatory observations collectively advocate against the unilateral classification of BrS as a monogenic disorder. Recent endeavors encompassing genome-wide association meta-analyses employing polygenic risk scores have unveiled the influence of multiple single nucleotide polymorphisms in shaping the Brugada phenotype [21].

## 3. Histological Findings

Histological studies have revealed that microstructural abnormalities of the extracellular matrix, particularly fibrosis, are observed in BrS [22]. Longitudinal observations using cardiac magnetic resonance imaging have shown the development of new late gadolinium enhancement within the subepicardial myocardium of the right ventricle, accompanied by progressive right ventricular systolic dysfunction over time [23]. The findings suggested that in some cases of BrS, there is a progressive course with focal fibrosis, especially in the right ventricle [23]. Furthermore, we documented the advancement of conduction disorders over time using surface ECG of symptomatic patients [24]. Herein, a hypothesis arises that Brugada-type ECG is a marker of structural heart disease. In fact, we reported that some patients with arrhythmogenic right ventricular cardiomyopathy present Brugada-type ECG patterns, and these ECG characteristics predict unfavorable outcomes for them [25]. Hoogendijk MG. et al. advocate the current-to-load mismatch theory, suggesting that localized excitation failure from the sub-endocardium to the sub-epicardium and/or delayed excitation from an adjacent site cause J-ST elevation in an experimental and computational study (Figure 1A) [10]. These findings support a conduction disorder hypothesis.

Nonetheless, there remains a conspicuous absence in our understanding regarding the rationale behind the exacerbation of J-ST elevation by well-acknowledged environmental factors such as vagal response, fever, oral glucose tolerance, and a satiated stomach, as documented in previous studies [26,27,28,29]. The underlying causes for the dampening effect of low-dose isoproterenol on electrical storms of VF remain shrouded in ambiguity [30]. Furthermore, it is noteworthy that arrhythmias linked to conduction anomalies typically present as monomorphic ventricular tachycardia. However, in the context of BrS, almost all instances of tachy-ventricular arrhythmias manifest as VF, posing an intriguing question concerning whether BrS can be solely attributed to a conduction disorder.

Although a recent investigation has documented a mutation in the thermosensitive segment of SCN5A, the precise connection between vagal activity and J-ST elevation remains enigmatic [31]. Notably, a degree of overlap was discerned between SCN5A mutations and structural cardiac malformations in select cases of BrS; however, it merits attention that the majority of BrS patients do not display discernible structural abnormalities via widely employed imaging modalities [32]. An emerging hypothesis posits that SCN5A mutations predominantly engender localized conduction perturbations at a functional rather than structural level [33]. For further clarity on the pathogenesis, Figure 1B provides an illustrative schematic depicting genetic and histological findings.

## 4. Electrophysiological Findings

While the epicardium of the right ventricle has historically been recognized as a pivotal locus, the establishment of a direct mapping technique for patients has hitherto remained elusive [34]. However, Nagase S. et al. introduced an innovative methodology wherein an electrical guidewire is intricately threaded into the conus branch of the right coronary artery, thereby facilitating the direct acquisition of epicardial electrocardiograms [35]. Their findings revealed that localized late potentials, which occur subsequent to the termination of the QRS complex, can be meticulously recorded. Notably, the administration of a sodium channel blocker was observed to extend the duration of these late potentials. Consequently, late potentials within the epicardium emerged as a pivotal hallmark within the spectrum of Brugada-type electrocardiogram patterns.

Recent strides in electrophysiological methodologies have heralded a transformative era in our understanding of BrS. Firstly, the advent of three-dimensional mapping systems has bestowed the capability to furnish meticulous anatomical and electrophysiological insights spanning the entirety of the cardiac organ. Secondly, the establishment of a percutaneous epicardial approach technique has opened new avenues for investigation [36]. Presently, catheter ablation aimed at targeting late potentials residing within the epicardium has ascended to prominence as a standard therapeutic modality for BrS [37,38]. However, it is strikingly evident that our comprehension of the underlying mechanisms governing these aberrant potentials remains conspicuously limited.

Experimental models have well-described that abnormal repolarization contributes to delayed and fractionated potentials [39]. One of the advantages of this theory is that it can explain the reasons for VF occurrence via phase 2 reentry. Phase 2 reentry is a theory proposed as a mechanism for arrhythmogenesis, where electrotonic current propagates from a region with a spike-and-dome action potential in the endocardium to a region with a loss of dome in the action potential in the epicardium [40]. However, the tone of recent papers primarily discusses the cause of abnormal potentials as conduction abnormalities using epicardial electrophysiological mapping. In the epicardial mapping of patients with BrS, extra stimulation results in the prolongation of delayed potentials or localized conduction block, in other words, excitation failure [41]. We also reported a case in which Brugada-type J-ST elevation was observed in unipolar electrodes, which were set with the same band-pass filter as surface electrocardiograms, along with a prolongation of the duration of bipolar late potentials [42].

The delayed and fractionated potentials observed in bipolar electrodes typically indicate slow or zig-zag conduction in structurally abnormal diseases [43]. A paper that focused on the local negative T-wave on unipolar electrodes demonstrated that the negative T-wave amplitude becomes shallower along with a decrease in bipolar late potentials amplitudes [44]. It concluded that local conduction heterogeneity affects J-ST and negative T-wave formation, which is a necessary condition for coved-type Brugada ECG [44]. Our case report also demonstrated that pilsicainide induced convex-type J-ST elevation, indicating the disappearance of negative T-waves, along with the shortening of bipolar delayed potentials [42]. This observation could be interpreted as a localized conduction block utilizing a sodium channel blocker. The hypothesis of conduction abnormalities is compelling.

Nevertheless, prior studies have primarily focused on establishing the association between J-ST elevation and conduction disorders, with little insight into the mechanisms underlying VF. A bipolar electrogram is conventionally derived by subtracting two unipolar electrograms acquired from sites that are typically in close proximity. Hence, delving into the analysis of unipolar electrograms becomes imperative to unravel the intricacies of bipolar electrograms and the mechanisms orchestrating ventricular arrhythmias. In the context of J-wave syndrome, a noteworthy case report centered on unipolar electrograms has postulated a phase 2 reentry theory as the instigator of premature ventricular contractions (PVCs), culminating in spontaneous VF [45].

**Figure 2:**
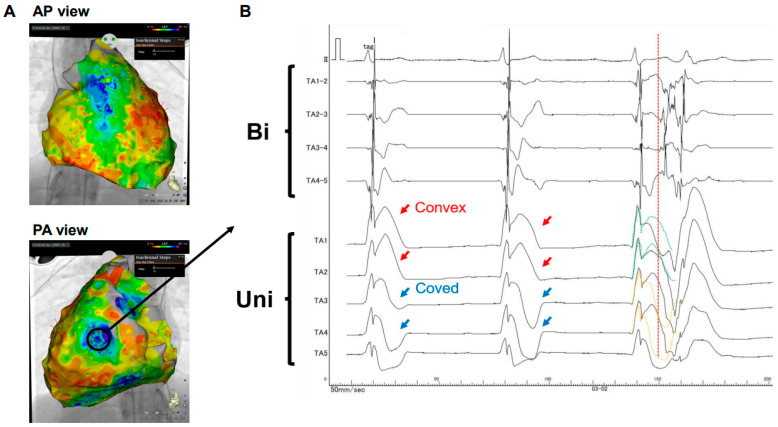
A case that may substantiate the influence of phase 2 reentry on premature ventricular contractions. (**A**) The local activation time map in the epicardium. Cool colors indicate the delayed excitation area. (**B**) Electrodes TA 1 to 5 represent unipolar electrodes, while electrodes TA 1–2, 2–3, 3–4, and 4–5 correspond to bipolar electrodes. Electrodes TA 1 and 2 registered convex-type J-ST elevation (indicated by red arrows), while electrodes TA 3 and 4 captured coved-type elevation (indicated by blue arrows). The occurrence of premature ventricular contractions, with the earliest activation site identified at TA 2–3 in bipolar electrodes, is indicated by the presence of a red dashed line. TA 2–3 delineated a transitional region between convex and coved type J-ST elevation patterns, implying that phase 2 reentry played a role in the occurrence of premature ventricular contractions. The green and yellow dashed lines represent the morphologies from one beat ago. Bi indicates bipolar electrodes; green lines: the convex morphologies; Uni indicates unipolar electrodes; and yellow lines: the coved morphologies.

Furthermore, our own encounter with an electrophysiological mapping case involving BrS facilitated by a state-of-the-art three-dimensional system yielded remarkable insights. In this case, a PVC was identified as the harbinger of spontaneous VF, with its origin traced to the epicardium (Figure 2). Notably, this event coincided with a confluence of convex- and coved-type J-ST elevations recorded in unipolar electrodes situated along the lateral aspect of the left ventricle (denoted by the blue markers in Figure 2A). These elevations corresponded to the region manifesting the most delayed excitation (as depicted by the cooler coloration in Figure 2A). A similar pattern was also observed along the epicardium of the right ventricular outflow tract, as depicted in Figure 2A.

Of paramount significance, we successfully identified the earliest site of excitation timing in relation to PVC. This pivotal observation was made at the unipolar electrode positioned precisely at the transitional interface between convex-type (emphasized by the red arrows in Figure 2B) and coved-type (as denoted by the blue arrows in Figure 2B) J-ST elevations. This compelling finding is in profound accordance with the underpinning principles of the phase 2 reentry theory, which fundamentally revolves around the concept of dispersion within the epicardial realm, as distinct from transmembrane effects (refer to Figure 1A). Consequently, we are now suitably positioned to advance the hypothesis that ventricular tachyarrhythmias may be triggered by repolarization anomalies, particularly the pronounced heterogeneity observed in the action potentials manifesting within the epicardial milieu.

## 5. Proposed Mechanisms of BrS

**Figure 3:**
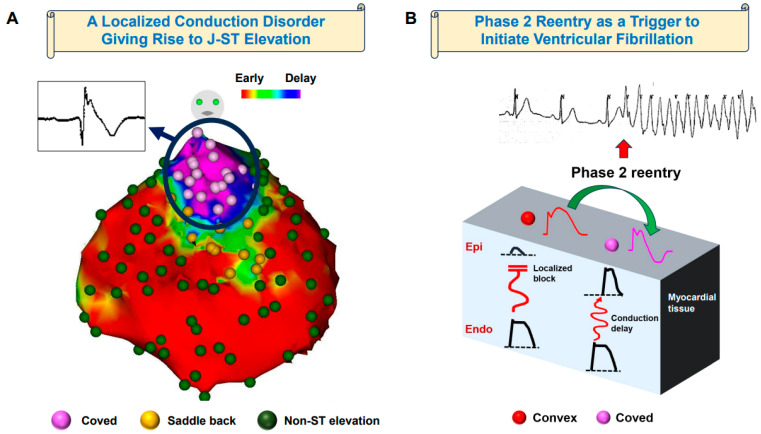
Proposed mechanisms of Brugada syndrome. (**A**) A local activation time map of the epicardium in patients with Brugada syndrome who have experienced episodes of ventricular fibrillation storms. The sector demonstrating the most pronounced delay in excitation exhibited the distinctive coved-type J-ST elevation pattern, as indicated by the presence of pink markers on the unipolar electrodes. In contrast, the region with a less pronounced delay displayed the saddle-back type of elevation, represented by yellow markers. These observations strongly imply that conduction irregularities play a significant role in the genesis of J-ST segment elevation. (**B**) A three-dimensional conduction model for Brugada syndrome. In the milieu of excitation conduction spanning from the endocardium to the epicardium, the presence of localized conduction impediments serves as a significant catalyst in the genesis of convex-type J-ST elevation (represented by red markers). Conversely, instances of conduction sluggishness give rise to the configuration typified by coved-type J-ST elevation (indicated by pink markers). The presence of disparate activation potential durations across various epicardial zones contributes substantively to the phenomenon of phase 2 reentry, thereby intricately interplaying in the genesis of ventricular fibrillation episodes.

Based on these findings, it is imperative to analyze the mechanisms of J-ST elevation with a negative T-wave in surface ECG independently from those associated with ventricular tachyarrhythmias in a distinct manner. The Brugada-type ECG should manifest as a result of localized conduction disorders, which are influenced by gene mutations characterized not by a single monogenic factor but rather by multiple single nucleotide polymorphisms. These manifestations may also be associated with localized structural heart diseases. However, the occurrence of PVC-initiated spontaneous VF should manifest due to the heterogeneity of action potentials within the epicardium. The mechanisms we propose are depicted in Figure 3.

## 6. Future Outlook

Recent genetic, histological, and electrophysiological findings have been progressively elucidating the mechanisms of Brugada-type ECG and spontaneous ventricular tachyarrhythmias. However, risk stratification and therapeutic strategies of the primary prevention for asymptomatic patients remain unclear. Cutting-edge technologies, such as whole-genome sequencing to evaluate the effects of multiple single nucleotide polymorphisms, in situ hybridization of epicardial tissue to screen for the messenger RNA expression related to ion channels, or electrophysiological studies focusing on unipolar electrodes to better understand the mechanisms of BrS-type ECG, as well as standardizing electrophysiological study protocols, may serve as pivotal tools for achieving breakthroughs. Further fundamental and clinical investigations are warranted to understand the complexity of BrS, which will lead to the establishment of risk stratification.

## 7. Conclusions

BrS represents a conceptual framework encompassing a myriad of common symptoms, albeit with an etiology that remains elusive. Within this construct, BrS emerges as a diverse spectrum of channelopathies characterized by robust genetic underpinnings, as well as structural abnormalities, predominantly featuring fibrosis within the epicardial domain. It is imperative to underscore the imperative connection between conduction aberrations and the prominent J-ST elevation evident in the right precordial leads.

The observed electrophysiological heterogeneity, particularly within the three-dimensional myocardial tissue, notably the right ventricular outflow tract, is poised to wield significant influence over the incidence of VF. The prospect of catheter ablation directed at the Brugada substrate presents the potential to harmonize this electrophysiological diversity, thereby serving as a means to mitigate the occurrence of VF events [38]. A comprehensive continuum of further investigations, incorporating genetic profiling, histological evaluations, and electrophysiological inquiries, holds promise for an enriched comprehension of the intricate mechanisms underpinning BrS.

## Data Availability

Not applicable.

## Abbreviations

BrS	Brugada syndrome
ECG	electrocardiogram
PVC	premature ventricular contraction
VF	ventricular fibrillation
